# Vortex clustering in trapped Bose-Einstein condensates

**DOI:** 10.1038/s41598-023-46549-3

**Published:** 2023-11-08

**Authors:** Thomas Easton, Marios Kokmotos, Giovanni Barontini

**Affiliations:** School of Physics and Astronomy, University of Birmingham, Edgbaston, Birmingham, B15 2TT UK

**Keywords:** Physics, Atomic and molecular physics, Ultracold gases

## Abstract

We numerically study the formation of vortex clusters in trapped Bose-Einstein condensates where vortices are initially imprinted in a line. We show that such a system exhibits a rich phenomenology depending on the distance at which the vortices are imprinted and their number. In particular we observe that it is possible to obtain systems of twin vortex clusters, twin vortex clusters with orbiting satellite vortices, and triplets of clusters. By using a clustering algorithm we are able to quantitatively describe the formation and dynamics of the clusters. We finally utilise an analytical model to determine the range of parameters for which the clustering occurs. Our work sets the stage for possible experimental implementations where the formation of vortex clusters and more exotic bound states of vortices could be observed.

## Introduction

The notion of vortices and vorticity is ubiquitous in physics, as it appears in classical physics, e.g., in hydrodynamics, gravitational physics, electromagnetism^1^ and non-linear optics^2^, as well as in in the quantum realm, through quantum turbulence and quantum hydrodynamics in superfluids^3^ and superconductors^4^. Bound structures such as vortex-vortex or vortex-antivortex pairs have been created in BECs^5–8^ and in ultracold Fermi gases^9^. In the optical regime, vortex cluster formations have also been demonstrated in microcavity exciton-polariton condensates^10^. The processes and parameters that lead to the formation of vortex clusters rather than, e.g., turbulence or disorder, are however less understood and investigated.

Atomic BECs are a good platform for the generation and study of vortex clusters and their dynamics due to their extensive controllability in the experiments^11^ and the wide availability of tools for numerical simulations both at zero^12^ and finite temperature^13,14^. Notably, in BECs vortices appear as localised topological defects which carry quantised angular momentum^15–17^. In trapped BECs, the vortex dynamics are dictated by density and phase gradients of the superfluid background. The gradient of the background density created by the trapping potential induces a precession of the vortex about the center of the trap. In addition, each vortex creates a velocity field that affects the motion of the other vortices similarly to the way seen in fluid physics. This can be seen as a vortex-vortex interaction that depends on the separation of the vortices as well as their topological charges^18^. It can lead to a rotational motion where the vortices rotate around each other, or a linear motion where the vortices travel in parallel. The combination of these effects is at the core of the formation of vortex clusters, turbulence or vortex lattices.

For what concerns vortex clusters, the many features of trapped BECs are ideal to gain precious insight, both in real experiments and in numerical simulations, on the dynamics of the clusters' formation and the conditions for their existence and stability. In this work, we numerically study the conditions under which linear vortex structures, imprinted onto a trapped BECs, can evolve into vortex clusters. The system under investigation has been chosen with an eye to possible experimental implementations, as vortices can be controllably imprinted on ultracold atomic samples^8,19^. Our configuration of choice is the case of simple one-dimensional linear structures which are symmetrical about the centre of the cloud. This layout has a symmetry that is expected to favour the formation of clusters, but also has the characteristic of being geometrically very different from clusters. This combination allows us to observe the dynamical formation of the clusters and determine under which initial imprinting conditions such dynamics happen. In particular, we study the clustering dynamics of arrangements of $N_v$ vortices arranged on a line passing through the centre of the cloud, with a symmetric uniform distribution of vortices on each side. While similar configurations in an anisotropic cloud have been considered in the past where the ordering of the linear layout is not destroyed^20^, our isotropic system gives rise to clusters with rich dynamics. With the aid of a clustering algorithm, we are able to identify when clusters are formed in our numerical simulations, and to determine their lifetime. Furthermore, we employ a simple analytical model to scan the space of parameters of our imprint pattern and understand which clusters configurations are accessible for a given initial configuration. With the same model, we finally demonstrate that the vortex clusters effectively behave like a single entity, namely like a highly charged vortex, with the charge given by the sum of the charges of the constituent vortices.

## The system

We numerically simulate the imprinting of vortices and the following dynamics using the Gross-Pitaevskii equation (GPE), that we write here in dimensionless form

1 $$\begin{aligned} i\partial _t \Psi (\vec {r},t)=\bigg (-\frac{1}{2}\nabla ^2 +V(\vec {r}) +\beta |\Psi (\vec {r},t)|^2\bigg )\Psi (\vec {r},t), \end{aligned}$$

where we have used the inverse of the harmonic oscillator frequency $\omega _{ho}=\lambda ^{1/3}\omega _\perp$, and the harmonic oscillator length $a_{ho}=\sqrt{\hbar /m\omega _{ho}}$, with *m* the mass of the atoms, as units of time and length. The interaction term is $\beta =4\pi a$, where *a* is the s-wave scattering length. Split-step methods are used to numerically solve the equation. For concreteness, we will consider $N=5\times 10^4$ $^{87}$Rb atoms trapped in a harmonic potential with trapping frequencies $\omega _x=\omega _y=\omega _\perp$ and $\omega _z=\lambda \omega _\perp$ in the radial and transverse directions respectively, with $\lambda =15$ and $\omega _\perp = 2\pi \times 20$ Hz. For this set of parameters the Thomas-Fermi radius is 11.8 while the healing length is 0.2. We numerically simulate our system on a $256\times 256\times 16$ grid with a resolution of $0.15\times 0.15\times 0.12$. The strong anisotropy between radial and transverse directions allows us to treat our system effectively as a 2D BEC where the vortices are constrained to move in the plane as their bending would be energetically unfavourable.

**Figure 1:**
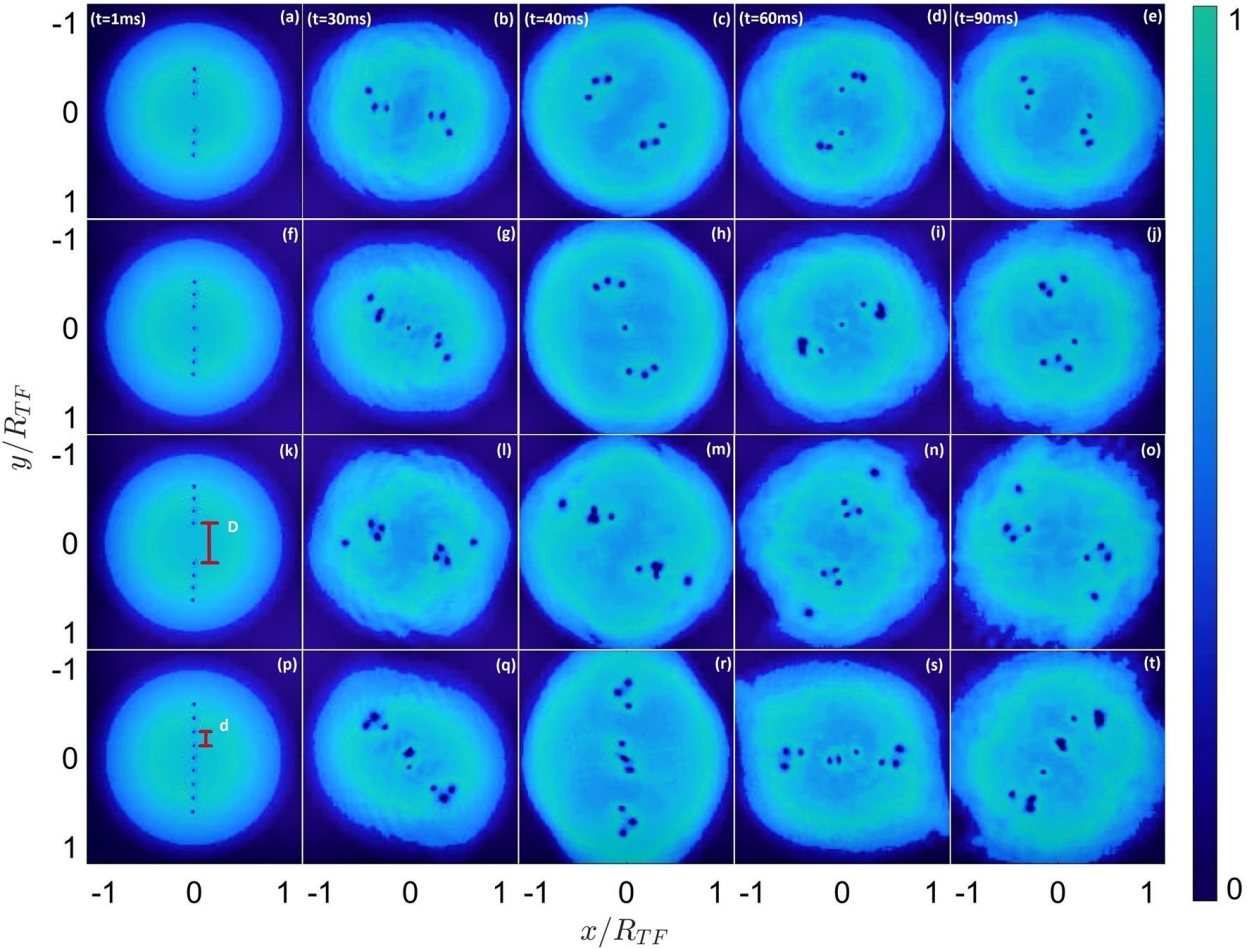
Snapshots of the column density in the *xy* plane over time. The evolution time for each panel is indicated in the top row. Distances are normalised to the Thomas-Fermi radius $R_{TF}$. The red segments in panels (k) and (p) correspond to *D* and *d* respectively, as defined in Sec. II. The colour scale corresponds to the column density value normalised to the maximum in each panel. The top row shows $N_v=6$ vortices with $D=0.414 R_{TF}$ and $d=0.138 R_{TF}$. The second row shows $N_v=7$ vortices with $D=0.48 R_{TF}$ and $d=0.138 R_{TF}$. The third row shows $N_v=8$ vortices with $D=0.44 R_{TF}$ and $d=0.138 R_{TF}$. The bottom row shows $N_v=9$ vortices with $D=0.274 R_{TF}$ and $d=0.154 R_{TF}$.

To initiate the dynamics that generates the vortex clusters, at $t=0$ we imprint on the condensate a spatially dependent phase profile of the form

2 $$\begin{aligned} \phi (x,y) = 2\sum _{n=1}^{N_v} s_n \text {tan}^{-1}\bigg (\frac{y-y_n}{\sqrt{(x-x_n)^2 +(y-y_n)^2}+x-x_n}\bigg ), \end{aligned}$$

where $s_n$ is the vortex charge for the *n*-th vortex and $(x_n,y_n)$ are the coordinates of the *n*-th vortex. We arrange a number of vortices, $N_{v}$, in two groups symmetrically along a common diameter of the cloud. Within each of these groups, we choose a constant nearest-neighbour distance, *d* (see Fig. 1p). The closest distance between the two groups is then parameterised by *D* (so, excluding the central vortex of the line in case of odd $N_v$, *D* is the distance between the two inner-most vortices) as depicted in the first column of Fig. 1. In the cases where there is an odd vortex number, we also include a vortex at the centre of the cloud. All vortex lines are parallel to the z-axis and have the same charge. The addition of one or more vortices of opposite charge favours the formation of vortex dipoles that travel outside the condensate, preventing the formation of the clusters.

## Vortex dynamics

As soon as the vortices are imprinted, they begin to precess around the centre of the cloud in a direction given by the sign of the imprinted topological charge (see Fig. 1). Because the precession frequency is an increasing function of distance from the centre of the cloud, the outer vortices rotate faster than the inner ones, leading to the line bending into an ‘s’-shape. Without vortex-vortex interactions, one would expect that this bending would become more and more exaggerated, leading the vortices to wind into a spiral configuration with each vortex remaining at its original radial distance from the centre of the cloud. However, the existence of vortex-vortex interactions, although repulsive, gives rise to dynamics that leads to the creation of vortex clusters. In particular, as the interaction scales inversely with the distance between vortices, the vortices tend to rotate around their closest neighbours, causing the linear structure to break into clusters, as shown in Fig. 1. Once formed, the vortex clusters rotate around their centroids, and continue to precess around the centre of the cloud. Typically, the arrangements of clusters remain well separated from one another and maintain the symmetry of the initial state, forming two identical antipodal clusters or two antipodal clusters and an additional one in the centre, depending whether $N_v$ is even or odd.

Depending on the number of vortices imprinted and the distances *d* and *D*, we observe a range of different clustering dynamics. An example of the simplest kind of dynamics is reported in the first row of Fig. 1. We observe this dynamics when $D\gg d$, therefore when the vortices are already imprinted into two fairly separated groups, and when $N_v$ is even. The dynamics that follows the imprinting essentially keeps the overall symmetry, and the two lines of vortices evolve into two separated clusters that orbit around their centroids. The inner cluster dynamics is quite complex, in the sense that the two clusters change their shape often, however, as we discuss later on, the average distance between the vortices in the cluster and the distance between the clusters do not substantially change. We observe similar dynamics when $D\gg d$ and $N_v$ is odd. An example is shown in the second row of Fig. 1. In this case, the central vortex remains stationary while the antipodal clusters form and begin to evolve as described previously. However, this central vortex will eventually be perturbed and move from the centre, usually joining one of the existing clusters (an example of this can be seen in Fig. 1i,j). Once the central vortex moves, the symmetry of the system breaks and the clusters are perturbed. The perturbed cluster ceases to be tightly bound and its dynamics is no longer a simple revolution around its centroid, while the centroid itself changes. The perturbation of the central vortex occurs due to the propagation of numerical errors and the fact that the vortex is in an unstable equilibrium. Such numerical errors reflect the real-life experiments where noise or quantum depletion can destroy the initial symmetry, phenomena that are not accounted for in the GPE.

The clustering dynamics becomes richer as $N_v$ increases. An example is shown in the third row of Fig. 1, with $N_v=8$ and $D\gg d$. In this case we observe that the dynamics that follows the imprinting generates clusters with a satellite vortex. These are formations of strongly-bound clusters that revolve around their centroids, similarly to what previously described, with an additional vortex that revolves around them. The satellite vortices are at a distance from the others that doesn’t allow them to be considered part of the cluster, nor to be considered as stray vortices. Another kind of clustering dynamics is shown in the fourth row of Fig. 1, for $N_v=9$ and $D\simeq 2d$. Here we observe the formation of three clusters. The third cluster forms around the central vortex and remains stable until it is perturbed by the other two (Fig. 1t).

As we discuss in Sec. V, we do not observe any clustering dynamics for small values of *D*, where the initial configuration of the imprinted vortices causes the trajectories to tangle and does not allow the formation of well separated clusters. We also do not not observe clustering for large values of *d*, where the vortices fail to capture one another in their velocity fields. Additionally, for the range of parameters that we simulated, we did not observe the onset of tight 4-vortex clusters.

## Cluster detection

**Figure 2:**
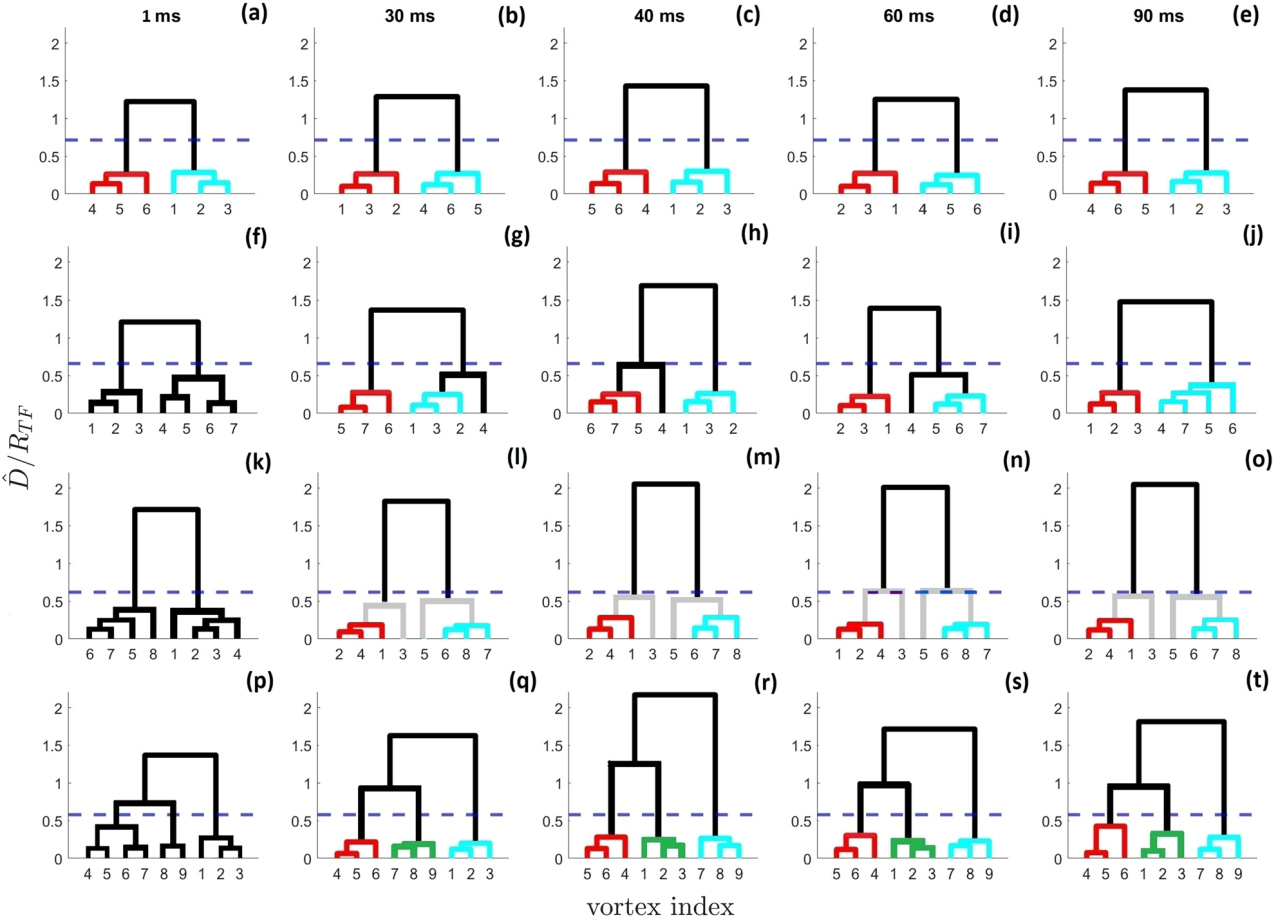
The figure shows the dendrogram for the hierarchical agglomerative algorithm for the same data as Fig. 1. The evolution time for each panel is indicated in the top row. The merging distances ${\hat{D}}$ computed by the clustering algorithm are normalised to $R_{TF}$. The horizontal dashed line in each panel corresponds to $\delta$. The first row corresponds to $N_{v}=6$ vortices. The algorithm detects two clusters of three vortices each, highlighted with different colours. The second row shows the case of $N_{v}=7$ vortices. The third row is for the case of $N_{v}=8$ vortices, where the grey branches identify the ‘satellite’, vortices. The bottom row is for the case of $N_{v}=9$ vortices, where three clusters are formed, identified with different colours.

Various algorithms and statistical methods have been used to devise clustering measures in a 2D Bose-Einstein Condensate in systems of 2D decaying quantum turbulence^21,22^ and low vortex-number systems^23,24^. To provide a quantitative description of the clustering dynamics and to determine when a cluster has been formed, we have applied the Hierarchical Agglomerative Clustering algorithm^25^. The quantitative definition of a cluster is indeed a common problem in statistical theory and its modern machine learning applications^25^, where clusters of similar objects are detected based on similarity of their features in a multi-dimensional space. In our system we seek clusters in a Euclidean two-dimensional geometry. The clustering algorithm detects clusters by analysing the positions of the vortices with no external input besides the definition of the algorithm’s metrics. The only precondition for a successful run of the algorithm is that we are able to accurately describe the trajectories of the individual vortices. This is a reasonable requirement for our case of interest that consists of vortices with well separated cores. The calculation of the vortex trajectories is achieved using a vision detection algorithm that identifies the movement of well separated regions of zero intensity in the images of the system evolution. We restrict the detection to a circular region of variable radius that encloses the imprinted vortices of interest. Vortices that appear at the edge of the condensate are ignored. This speeds up the trajectory calculation. For the few cases where the detection algorithm fails, we manually extract the locations of each vortex. Compared to other approaches that use a nearest neighbour approach^21,22^, the Hierarchical Clustering algorithm does not require manually setting hyper-parameter values in advance, such as the number of nearest neighbours or a cut-off distance that will be used during runtime, which can drastically affect the qualitative results. There is a threshold that is set in advance based on the uniform spatial distribution expected average distance, but this is not related to the algorithm itself. The algorithm does not rely on the vortex charge in order to detect clusters, but can be easily extended to the case of opposite charge.

The algorithm starts by treating each vortex as an individual cluster, and then it iteratively attempts to merge the clusters in larger clusters following a bottom-up approach, according to the Ward criterion^26^. The selection of the optimal pair to be joined at each step is based on the distance metric

3 $$\begin{aligned} {\hat{D}}=\sqrt{2\frac{n_r n_s}{n_r + n_s}} \Vert {\textbf {r}}_r - {\textbf {r}}_s \Vert , \end{aligned}$$

where $n_r$ and $n_s$ are the number of elements in each of two clusters *r* and *s* respectively that are under consideration. Similarly, ${\textbf {r}}_r$ and ${\textbf {r}}_s$ are the positions of their centroids. At each step, the clusters with the minimum pairwise distance are connected. The distance is also proportional to the square root of the harmonic mean of the number of elements in each of the two cluster to be joined. The clusters appear as distinct formations in the dendrogram output of the algorithm, see Fig. 2. A strongly bound cluster is characterised by a short merging distance and large difference to the next merging point in the dendrogram.

Each panel in Fig. 2 corresponds to the same panel in Fig. 1. The dashed horizontal lines correspond to the average inter-vortex distance for uniform distributions of vortices, which is $\delta = \sqrt{\pi /N_v} R_{TF}/2$, for the same sets of parameters. In general, clusters must satisfy ${\hat{D}}\ll \delta$. It is important to note that $\delta$ is not a parameter of the algorithm but only a reference point for our interpretation of the results compared to a purely random layout. The first row of Fig. 2 shows that when $D\gg d$ and $N_v$ is even, one can already consider the imprinted pattern as two separate clusters (highlighted with different colours). The following dynamics essentially does not alter the clusters that keep their intra- and inter- distances roughly the same as the initial pattern. The case with $D\gg d$ and $N_v$ odd is instead shown in the second row. After the initial imprinting, two three-vortex clusters are rapidly formed while the remaining seventh vortex orbits at a distance $\simeq \delta$ from one of the clusters, therefore we can’t consider it part of any cluster. The dendrogram remains essentially stationary until $t=90$ ms, where it shows that the seventh vortex is finally incorporated in one of the clusters.

The third row of Fig. 2 reports the case of $N_{v}=8$ vortices. The dendrogram shows that two three-vortex clusters are clearly formed at a distance well below $\delta$, while each of the remaining vortices have a merging distance to one of the clusters near $\delta$. The fact that the merging distance of the fourth vortex is close to $\delta$ confirms the fact that it can only be considered as a satellite vortex and not part of the cluster itself. Interestingly the cluster-plus-satellite formations remain stable and stationary for as long as we follow their dynamics. This is a phenomenon that could not be predicted a-priori and is only discovered by the algorithm. The bottom row of Fig. 2 finally reports the dendrograms for the case of $N_{v}=9$ vortices. Shortly after the imprinting, the algorithm clearly identifies three very tightly bound clusters that remain well separated and stationary for most of the dynamics. Towards $t=90$ ms we observe that the clusters start to slightly weaken their binding, and for longer times we observe that the clusters dissolve.

## Parameter space for cluster formation

As just discussed, the initial imprinting gives rise to a range of different dynamics depending on the number of vortices $N_v$ and the distances *d* and *D*. However, not every imprinting configuration generates stable or quasi-stable clusters. To explore the range of parameters that lead to the formation of clusters, we utilise an effective particle model. This approach allows us to scan the space of parameters more easily than GPE simulations. In this simplified model the vortices are treated as point-like objects in a 2D space. The use of such a model, which also excludes effects from vortex line curvature and effects of vortex-phonon coupling, is justified in a quasi-2D system such as the one we consider. The motion of each vortex can be ascribed to two constituent factors. The first is the precession induced by the inhomogeneous atomic density profile of the condensate, while the second is the effect of pairwise interactions between the vortices. The latter can be explained as the motion that is induced on every point of a vortex in a pair due to the fluid flow that is generated by the other vortex of the pair.

**Figure 3:**
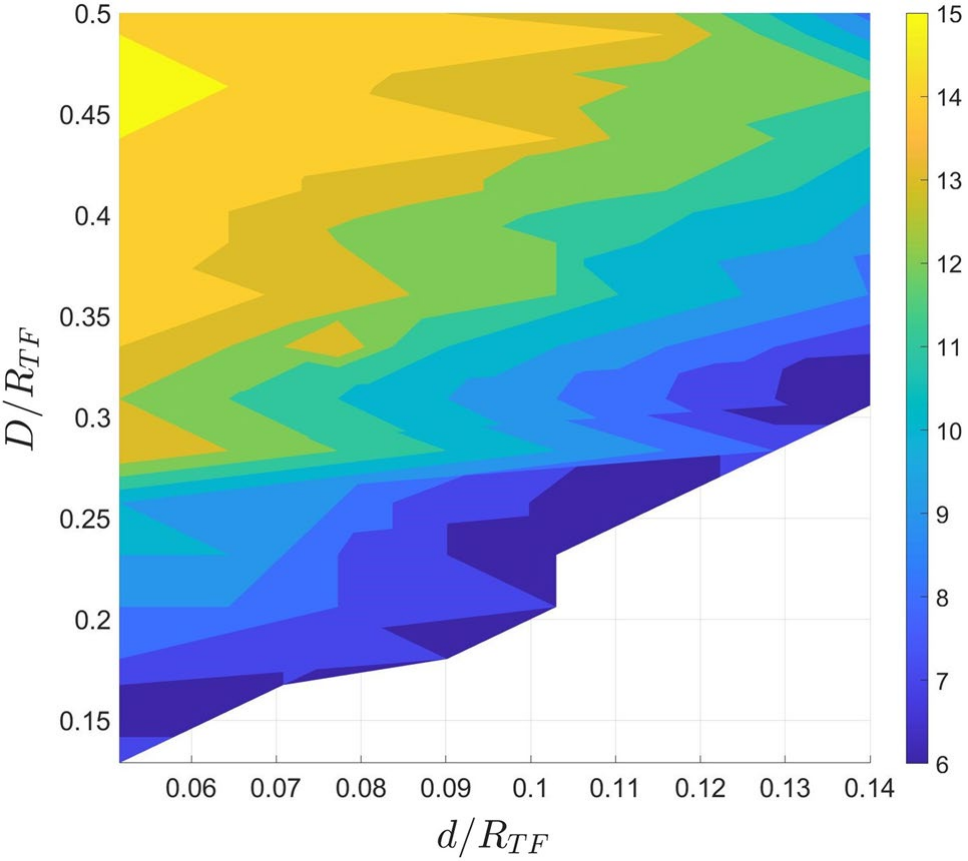
Maximum $N_v$ of imprinted vortices that can generate clusters as a function of *d* and *D*. The white region corresponds to configurations that either don’t produce clusters or can only produce clusters of two vortices. The non-smooth boundaries of the contours indicate the breakdown of the qualitative agreement between the ODE and GPE solutions.

As has been shown in Ref.^27^ the dynamics of the vortices can be expressed in the following system of first order ordinary differential equations (ODEs),

4 $$\begin{aligned} \dot{w_k}=i w_k \Bigg (\Omega _p \frac{s_k}{1-|w_k |^2} + \Omega _I \sum _{j\ne k}{\frac{s_j}{w_j^* w_k-|w_k |^2}}\Bigg ), \end{aligned}$$

with $s_k$ the topological charge of the vortex ($=\pm 1$), $w_k=z_k/R_{TF}$ the position of the *k*-th vortex, written as a complex number (i.e. $z_k=x_k+iy_k$), normalised to the Thomas-Fermi radius, $R_{TF}$ of the cloud. The first term in Eq. (4) corresponds to the precession of the vortex around the cloud while the second term corresponds to the interactions. The two frequencies controlling the relative strengths of these two effects are given by Ref.^17^,

5 $$\begin{aligned} \Omega _p= & {} \frac{(3+\frac{1}{5\lambda ^2}) \hbar }{2 m R_{TF}^2} \text {ln}\bigg (\frac{R_{TF}}{\xi }\bigg ), \end{aligned}$$

6 $$\begin{aligned} \Omega _I= & {} b\frac{\hbar }{2m R_{TF}^2}. \end{aligned}$$

where $\xi$ is the healing length, and *b* is an empirically determined parameter which modifies the strength of interactions.

We implemented Eq. (4) in a numerical ODE solver, using a ratio of $\Omega _p / \Omega _I \approx 9$, corresponding to the same values as simulated using the GPE. By comparing the dynamics observed in the ODE solver to those obtained by the numerical solution of the GPE, we found that the rotation period of the vortex clusters matched when $b=1.35$. This agrees with the value used in^27^.

By running simulations of the ODE system for various values of the *D*, *d* and $N_v$ parameters, we extract a clear region in which clustering occurs, shown in Fig. 3. Figure 3 shows the maximum number of vortices that can be imprinted, which generates clusters. For every $N_v$ smaller than this maximum value, we observe cluster formation. The configuration space of Fig. 3 shows that there is a strong relationship between the distance parameters *D* and *d* -according to which the initial imprinting of the vortices is configured- and the number of vortices that can cluster. We also observe good agreement between the ODE and the GPE solutions on whether clustering is observed for different values of *d*, *D* and $N_v$, except in cases which are very close to the boundary between clustered and unclustered states. As discussed, different initial *D* and *d* parameters lead to different cluster formations. We observe that a relatively large *D* value compared to *d* always lead to distinct clusters, even when the number of vortices is as high as 15. For odd and large number of vortices, we observe the formation of a central cluster in addition to the antipodal ones. As expected, for lower values of *D* and higher values of *d* the formation of clusters is hindered, and more complex dynamics occur.

## Analytical model for cluster dynamics

By treating the clusters as the objects of interest, we can extend the model developed above. This further simplifies the system by reducing the degrees of freedom but keeping the salient features of the system dynamics. In order to make a model of the dynamics of the clusters as opposed to their constituent vortices, we should first check whether the internal dynamics of a vortex cluster are important. In fact, in the case of a cluster of vortices in the absence of any external vortices or precession effects due to the shape of the cloud, it can be shown that the centroid position of any group of vortices with the same topological charge remains fixed.

**Figure 4:**
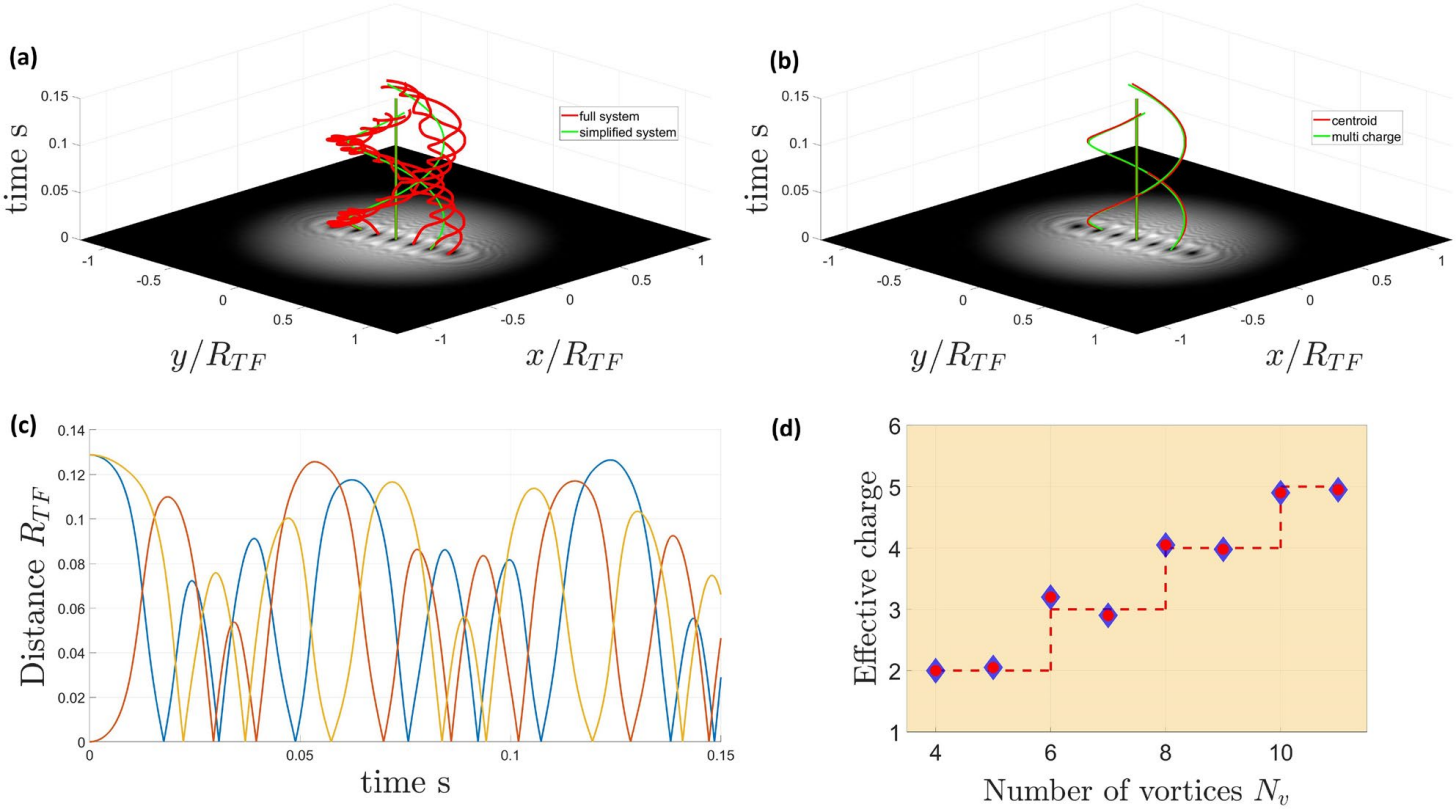
Comparison of the period of revolution and the dynamics of the clusters against the period of revolution of the composite object in the simplified system leads to an effective charge for the composite object for which a similar period is observed between the two systems. (**a**) Shows the trajectories of the original (full) system consisting of 7 vortices (in red) and the trajectories of the equivalent simplified system (in green). (**b**) There is a good agreement of the dynamics between the simplified system (green trajectories) and the centroids of the original system (red trajectories). (**c**) The distance of the individual trajectories of the full system to the trajectory of the multiply-charged vortex in the simplified system (motion in the reference frame of the multiply-charged vortex). Results for one of the clusters are shown. For the duration of the simulation the central vortex is unperturbed. For the full duration of the simulation, the distance of the individual vortices to the multiply-charged vortex is lower than their initial imprinted distance indicating that the cluster remains stable. (**d**) Shows the agreement of the effective charge of the simplified ODE system (composite object) on the vertical axis and the sum of the vortex charges in the cluster of the full ODE system. For a system with $N_{v}$ vortices the effective charge agrees with the sum of the individual cluster (the integer part of ${N_v/2}$).

We define the cluster position to be its centroid,

7 $$\begin{aligned} C=\frac{1}{N}\sum _{k=1}^N w_k. \end{aligned}$$

Then, using Eq. (4), the equation of motion for this centroid is,

8 $$\begin{aligned} \dot{C}=\frac{i \Omega _I s}{N}\sum _{k=1}^N\sum _{j=1}^N {\left\{ \begin{array}{ll} 0 &{}j=k\\ \frac{1}{(w_j-w_k)^*} &{}j\ne k, \end{array}\right. } \end{aligned}$$

where we set $\Omega _p=0$ to remove the effects of precession due to the cloud density, and only summed over the *N* vortices within the cluster, thus eliminating the effects of interactions with the rest of the system. Clearly, from the anti-symmetry under dummy-index interchange, this is gives $\dot{C}=0$. In other words, an isolated cluster of vortices on a flat background won’t move on it’s own, in the same way that a single isolated vortex wouldn’t.

It follows from this that if we consider small clusters such that the effects of their differing positions in the velocity fields due to external vortices and the shape of the cloud is negligible, we may discard all of the internal dynamics of a cluster and consider it to be a single object, with its position taken to be the centroid of its constituent vortices.

The velocity of a vortex cluster centroid due to external sources such as those produced by the cloud shape or the effects of other vortices and clusters is equal to the mean of the velocity of its constituent vortices, which in turn depend only on their positions. As we consider small clusters, we can take the velocity of a vortex cluster to be the same as that of a single constituent vortex at the centroid position.

The effect of the cluster on an external vortex (or vortex cluster) is the sum of the velocity fields of all of its constituent vortices. If we assume that all external vortices have distances from the cluster much larger than the size of the cluster, we can simply treat the cluster as a vortex with a charge equal to the total charge of its constituent vortices.

These observations allow us to generalise Eq. (4) to one describing the dynamics of clusters of 1 or more vortices,

9 $$\begin{aligned} \dot{w_k}=i w_k \Bigg (\Omega _p \frac{s_k}{1-|w_k |^2} + \Omega _I \sum _{j\ne k}{\frac{s_j n_j}{w_j^* w_k-|w_k |^2}}\Bigg ), \end{aligned}$$

where the positions $w_i$ and charges $s_i$ now refer to the centroid positions and topological charges per vortex of a cluster, and $n_i$ gives the number of vortices found in the cluster labelled *i*. This model is only applicable for clusters formed from vortices of the same charge, however, due to the instability of vortices with absolute charges greater than one, this is the case of primary interest.

To test the validity of this model, it was implemented in the same numerical ODE solver as Eq. (4). The period of rotation was used as the comparison factor between the simulations of the two models, and it was found to be in a really good agreement (see Fig. 4). Specifically, for the case of even $N_v$ the line segments consisting of $\lfloor {N_{v}/2} \rfloor$ vortices, when clustered as per the configuration space of Fig. 3 were replaced by an object with an effective charge that turns out to be in a very good agreement with the expected sum of charges in the cluster. For the cases of an odd number of vortices, the central vortex remains unchanged while the symmetrical line segments with respect to the cloud are replaced with an object of an effective charge. The effective charges in both cases show a very good agreement with the expected total sum of charges in a cluster. In systems which are large enough to support multiple levels of length scale separation, we expect that one may observe this clustering process occur recursively, i.e. if there are many clusters which are well separated, they may form ‘superclusters’ in which each small cluster acts in the way that a single vortex does in a normal cluster. With enough length scale separation, this process may continue for multiple levels, however the ratio of cloud size to healing length would have to be very large to support enough clusters with enough distance between them, and the initial arrangements which would form these superclusters is not obvious.

## Conclusion

In conclusion, we have numerically and analytically studied the clustering of a set of vortices initially imprinted in a line on a trapped atomic Bose-Einstein condensate. This arrangement was chosen with an eye to possible experimental implementations, and to facilitate the clustering dynamics. We observed that for different values of the distance between vortices and between groups of vortices, a rich dynamics is generated. The symmetry of the setup favours the generation of twin vortex clusters orbiting around the centre of the cloud. When an odd number of vortices is imprinted we observe the formation of vortex clusters triplets or of twin vortex clusters with a single vortex in between. For higher numbers of vortices, we also observed the formation of twin vortex clusters with satellite vortices orbiting around them. With the aid of a hierarchical agglomerative clustering algorithm, we were able to quantitatively characterise the formation of clusters and understand the strength of their bond. We have shown that a simple analytical model can be used to efficiently explore the space of parameters and understand when clusters can form, and extended this model to show that vortex clusters behave in a very similar manner to highly charged vortices. Our work suggests that imprinting vortices in Bose-Einstein condensates could be a key enabler to understand the formation of vortex clusters and vortex molecules. Different initial patterns, including both vortices and antivortices with multiple topological charges, could lead to the formation of more exotic bound states, giant vortices^28^, and could shed some light on the transition between ordered and turbulent systems.

## Data Availability

The datasets used and/or analyzed during the current study are available from the corresponding author on reasonable request.
